# Effects of postpartum diastasis recti abdominis on multisite pelvic floor electromyography and anatomical structure: A cross-sectional study

**DOI:** 10.1097/MD.0000000000043067

**Published:** 2025-06-27

**Authors:** Feifei Fu, Shuang Zhang, Hongyun Zhang, Zhangbiao Xuan, Na Zhang, Zhenwei Xie

**Affiliations:** a Department of Gynecology, Affiliated Hospital of Shaoxing University, Shaoxing, Zhejiang, China; b Pelvic Floor Disease Diagnosis and Treatment Center, Women’s Hospital School of Medicine Zhejiang University, Hangzhou, Zhejiang, China; c Department of Ultrasound, Women’s Hospital School of Medicine Zhejiang University, Hangzhou, Zhejiang, China.

**Keywords:** anatomical structure of pelvic floor, diastasis recti abdominis, multisite surface electromyography, pelvic floor muscle, postpartum

## Abstract

This study evaluates the effects of postpartum diastasis recti abdominis (DRA) on multisite pelvic floor muscle electromyography (EMG) and pelvic anatomical structures, with the goal of informing targeted postpartum rehabilitation strategies. A total of 157 postpartum women who underwent follow-up examinations between April 2021 and August 2022 were included. EMG characteristics of pelvic floor muscles at multiple anatomical sites and structural changes were analyzed across groups. Additionally, correlations between the maximum inter-rectus distance and pelvic floor EMG parameters were assessed. Although the external anal sphincter showed significantly lower endurance contraction potential (ECP) in the non-DRA group compared to the DRA group (*P* < .05), multiple linear regression analysis indicated that DRA grouping was not an independent predictor of the ECP. In comparisons between mild and moderate-to-severe DRA groups, the anterior resting potential (ARP) of the puborectalis, pubococcygeus, and urethral sphincter muscles was significantly higher in the moderate-to-severe group (*P* < .05). Multivariate regression analysis further confirmed that DRA severity independently influenced ARP in the puborectalis (β = 9.344 μV, *P* = .018, 95% CI: [1.659, 17.030]), pubococcygeus (β = 8.601, *P* = .035, 95% CI: [0.621, 16.580]), and urethral sphincter (β = 7.903 μV, *P* = .015, 95% CI: [1.593, 14.213]). Weak correlations were observed between the maximum inter-rectus distance and both the ARP of the vaginal sphincter and the ECP of the external anal sphincter. No significant associations were found between DRA and three-dimensional ultrasound parameters of pelvic floor anatomy (*P* > .05). The severity of DRA affects EMG parameters related to muscle tone in specific pelvic floor muscles, particularly the ARP of the puborectalis, pubococcygeus, and urethral sphincter. Future rehabilitation strategies should take DRA severity into account and incorporate individualized factors such as delivery mode and parity to develop personalized interventions aimed at achieving more precise and effective postpartum recovery.

## 1. Introduction

The rectus abdominis plays a critical role in maintaining posture, facilitating trunk movements, stabilizing the pelvis, and supporting abdominal organs.^[1]^ Diastasis recti abdominis (DRA) occurs when sustained intra-abdominal pressure causes the linea alba to thin and widen, resulting in lateral separation of the rectus muscles from the midline. This condition is commonly observed in postpartum women, with an incidence ranging from 30% to 68%.^[2–6]^ Chronic and severe cases of DRA may lead to lower back pain, pelvic instability, and abnormal abdominal wall appearance.^[7,8]^ High-frequency ultrasound is the most widely used tool for assessing DRA and is considered the gold standard among noninvasive diagnostic methods.^[9,10]^ However, there is currently no unified diagnostic criterion at the national or international level.^[11]^ Most studies define DRA as an inter-rectus distance (IRD) >2 cm as measured by ultrasound.^[12,13]^

Pregnancy and childbirth are not only major causes of DRA but also significant risk factors for pelvic floor dysfunction (PFD).^[14,15]^ The pelvic floor muscles, which provide structural support for pelvic organs, may become weakened, leading to symptoms such as urinary incontinence and pelvic organ prolapse.^[16]^ Recent research suggests a synergistic relationship between the abdominal wall and pelvic floor. Structural impairment of the rectus abdominis may disrupt intra-abdominal pressure regulation and coordinated muscle activation, thereby indirectly affecting pelvic floor function.^[17–19]^ Despite growing interest, current studies on the relationship between DRA and pelvic floor muscle electromyographic (EMG) activity remain limited and yield inconsistent results, which limits their clinical applicability in postpartum rehabilitation.

We employed a novel airbag-type stretchable electrode array, which can cover various pelvic floor muscle groups and enables real-time acquisition of multichannel electromyographic signals.^[20–23]^ This system was integrated with three-dimensional perineal ultrasound to dynamically monitor pelvic floor structural movement, offering a comprehensive evaluation of muscle function. This study aims to investigate the electromyographic characteristics and anatomical changes of pelvic floor muscles in postpartum women with varying severities of DRA, using multisite surface electromyography (sEMG) and three-dimensional ultrasound. The findings are intended to inform the development of more targeted and effective postpartum rehabilitation strategies.

## 2. Materials and methods

### 2.1. Participants

**Figure 1.**
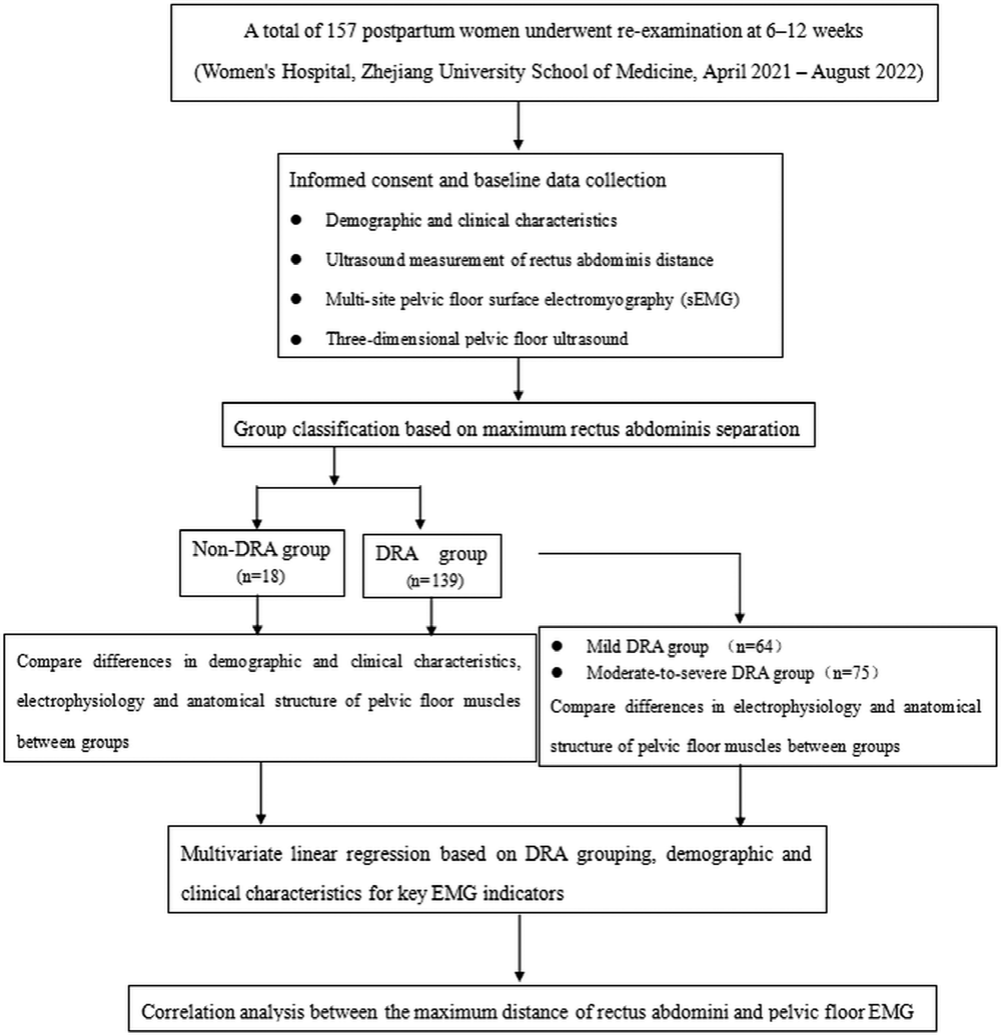
Flowchart of patients included in the study.

A total of 157 parturients who gave birth at the Women’s Hospital School of Medicine Zhejiang University and underwent postpartum reexamination from April 2021 to August 2022 were recruited as study subjects. The maternal age ranged from 21 to 42 years, with a median age of 31 years. There were 98 cases of vaginal delivery and 59 cases of cesarean section; 123 were primipara and 34 were multipara. In 157 cases, the incidence of DRA was 88.5% (Fig. 1).

Inclusion criteria: (1) Postpartum follow-up was performed for the first time between 6 and 12 weeks postpartum, and pelvic floor rehabilitation and rectus abdominis rehabilitation had not been performed before; (2) No vaginal bleeding or serious postpartum complications; (3) Those who completed rectus abdominis ultrasound, pelvic floor three-dimensional ultrasound, and multisite pelvic floor electromyography assessment.

Exclusion criteria: (1) Previous history of radical pelvic surgery or pelvic floor surgery; (2) Patients with malignant tumors and neurological diseases; (3) Trunk malformations such as severe scoliosis; (4) Patients with a history of increased abdominal pressure due to long-term constipation and coughing; (5) Lochia not clean or in the acute inflammatory phase of the urogenital tract; (6) Other systemic diseases that cannot be controlled.

This study was approved by the Ethics Committee of the Women’s Hospital School of Medicine Zhejiang University (No. [2019] Ethical Review Department No. [067]). Written informed consent was obtained from all patients.

### 2.2. Demographic and clinical characteristics collection

Demographic and clinical characteristics were collected from women giving birth, including age, height, mode of delivery, number of deliveries, gestational weeks, weight gain during pregnancy, body mass index before and during delivery, birth weight of the newborn, macrosomia, episiotomy and forceps delivery during delivery, episiotomy, history of macrosomic delivery, history of multiple pregnancies, gestational diabetes mellitus, PFD, history of abdominal surgery, etc.

### 2.3. Rectus abdominis distance measurement

Ultrasound measurements were conducted by a single experienced sonographer using the Mindray Resona 8S system with an L14-5WU linear probe (4.0–14.0 MHz). Participants lay in a supine position with the abdomen exposed from the xiphoid process to the symphysis pubis. Legs were flexed at approximately 90°, with soles flat on the table. At the end of expiration, IRD was measured at 4 standardized midline points—2 above and 2 below the umbilicus. Each site was measured 3 times, and the mean value was used. Based on criteria by Ranney et al^[24]^ and international guidelines, DRA was diagnosed if the IRD exceeded 2 cm at rest at any site. DRA severity was further classified by maximum IRD as follows: <3 cm as mild, 3 to 5 cm as moderate, and >5 cm as severe. Moderate and severe cases were combined as the moderate-to-severe DRA group.

### 2.4. Pelvic floor three-dimensional ultrasonic detection

Three-dimensional pelvic floor ultrasound was performed using the Mindray Resona 8S system with a 3 to 10 MHz volume probe. All scans were conducted by the same experienced sonographer. Prior to imaging, participants were trained in the Valsalva maneuver. Patients were placed in the bladder lithotomy position, with the posterior inferior margin of the pubic symphysis as the reference line. At rest and during Valsalva, the vertical distances from the lowest points of the bladder neck, external cervical os, and rectal ampulla to the reference line were measured. The levator hiatus area was also assessed during Valsalva. All measurements were performed 3 times and averaged. Organ mobility was calculated as the difference in position before and after the Valsalva maneuver.

### 2.5. Pelvic floor surface electromyography assessment

Flexible multichannel electrodes independently developed by our team (patent nos. CN201910419683.7, CN201910594638.5, ZL202110885418.5) were used to evaluate multisite sEMG signals of the pelvic floor and to capture electrophysiological activity from individual pelvic floor muscles. Prior to the assessment, patients were instructed to empty their bladders and assume a 120° semi-recumbent position with knees flexed, hips abducted and externally rotated, to ensure pelvic floor relaxation. Standardized instruction was provided to ensure proper pelvic floor muscle activation while avoiding the engagement of abdominal, gluteal, and medial thigh muscles. Flexible vaginal electrodes were then inserted and inflated to anchor the sensors firmly against the vaginal wall. Patients were guided to perform anal contractions according to the Glazer evaluation protocol. sEMG signals were collected from 6 pelvic floor muscles: the puborectalis, pubococcygeus, iliococcygeus, vaginal sphincter, urethral sphincter, and external anal sphincter. The following electrophysiological parameters were recorded:

1. Anterior resting potential (ARP) and coefficient of variation of ARP: Measured during a 60-second pre-contraction relaxation phase. The mean amplitude and its variability were used to assess resting pelvic floor muscle tone.
2. Rapid contraction potential: Derived from 5 maximal rapid contractions, each spaced 10 seconds apart. The peak EMG amplitude was averaged to evaluate fast-twitch muscle function.
3. Tonic contraction potential: Recorded during 5 sustained contractions, each lasting 10 seconds with a 10-second rest in between. The average EMG amplitude reflected the combined function of both fast- and slow-twitch fibers.
4. Endurance contraction potential (ECP): Collected over a continuous 60-second contraction. The mean EMG amplitude was calculated to evaluate slow-twitch muscle endurance.
5. Posterior resting potential and coefficient of variation of posterior resting potential: Measured during a 60-second post-contraction relaxation period, assessing the recovery capacity of pelvic floor muscles.
6. RMS ratio in the first 10 seconds after endurance contraction: This ratio compares the mean EMG amplitude of the last 10 seconds to the first 10 seconds of the 1-minute endurance phase, serving as an indicator of fatigue in type I muscle fibers.

### 2.6. Statistical analysis

Statistical analyses were performed using SPSS version 23.0. The normality of the data was assessed using the Shapiro–Wilk test. Continuous variables with a normal distribution were expressed as mean ± standard deviation ($\bar{\chi }\pm s$), and group comparisons were conducted using the independent-samples *t* test. For non-normally distributed data, values were presented as median and interquartile range [M (P25, P75)], and the Mann–Whitney *U* test was applied. Categorical variables were reported as frequencies (n) or percentages (%), and comparisons between groups were made using 2-tailed tests. Pearson correlation analysis was used for normally distributed data, while Spearman rank correlation analysis was applied to non-normally distributed data. Multiple linear regression analysis was conducted to assess the independent effects of selected factors on electromyographic outcomes. A 2-sided *P*-value < .05 was considered statistically significant.

## 3. Results

### 3.1. Comparison of demographic, clinical, and electrophysiological characteristics between non-DRA and DRA groups

There were no significant differences in baseline demographic or clinical characteristics between the non-DRA and DRA groups (*P* > .05) (Table 1). In the comparison of pelvic floor muscle electromyographic characteristics, the ECP of the external anal sphincter was significantly lower in the non-DRA group than in the DRA group (*P* < .05). No significant differences were observed in the electrophysiological parameters of other pelvic floor muscles between the 2 groups (*P* > .05).

**Table 1:** Comparison of demographic and clinical characteristics between non-DRA group and DRA group.

	Non-DRA group (n = 18)	DRA Group (n = 139)	*P*
Age (yr)	30.44 ± 3.65	31 (28, 33)	.709
Height (m)	1.59 ± 0.04	1.60 ± 0.05	.437
Week of gestation at delivery (wk)	38.33 ± 1.41	39 (38, 40)	.415
Pregnancy weight gain (kg)	13 (10.75, 15)	13 (10.70, 15)	.344
BMI at delivery (kg/m^2^)	26.84 ± 3.26	26.56 ± 3.19	.726
Prepregnancy BMI (kg/m^2^)	22.13 ± 3.62	20.89 (19.38, 22.92)	.570
Number of deliveries	1.16 (1.00, 1.00)	1.24 (1.00, 1.00)	.563
Neonatal birth weight (g)	3116.11 ± 429.04	3223.37 ± 463.87	.354
Macrosomia (n)	0	4	1.000
Mode of delivery (n)			
Vaginal delivery	12	86	.693
Obstetric forceps for assisted labor	1	9	1.000
Perineotomy	5	41	.880
Perineal laceration	7	35	.341
Cesarean section	6	53	.693
History of multiple pregnancies (n)	0	7	1.000
History of macrosomia delivery (n)	0	2	1.000
Combined PFD (n)	4	23	.788
History of abdominal surgery (n)	6	30	.413
Gestational diabetes mellitus (n)	2	26	.642

DRA = diastasis recti abdominis, PFD = pelvic floor dysfunction.

### 3.2. Comparison of electrophysiological characteristics of pelvic floor muscles between mild and moderate-to-severe DRA groups

The moderate-to-severe DRA group demonstrated significantly higher ARP values in the puborectalis, pubococcygeus, vaginal sphincter, and urethral sphincter muscles compared to the mild DRA group (*P* < .05) (Table 2). No significant differences were observed in the electrophysiological parameters of the remaining pelvic floor muscles between the 2 groups.

**Table 2:** Comparison of ARP values in 4 pelvic floor muscles between mild and moderate-to-severe DRA groups.

Muscle	Myoelectric parameters	Mild DRA group (n = 64)	Moderate-to-severe DRA group (n = 75)	*P*
Puborectalis	ARP (μV)	19 (10.17,3 8.58)	28.08 (13.70, 44.75)	.048
Pubococcygeus	ARP (μV)	19.99 (10.48, 38.79)	27.49 (16.63, 48.98)	.045
Vaginal sphincter	ARP (μV)	6.07 (3.22, 12.29)	8.81 (6.31, 17.23)	.008
Urethral sphincter	ARP (μV)	17.39 (10.13, 30.49)	25.54 (14.98, 37.64)	.038

ARP = anterior resting potential, DRA = diastasis recti abdominis.

### 3.3. Comparison of pelvic floor anatomical structure among groups

Three-dimensional pelvic floor ultrasound was used to assess anatomical features in women from both the non-DRA and DRA groups, as well as between the mild and moderate-to-severe DRA subgroups. No significant differences were observed in bladder neck position or mobility before and after the Valsalva maneuver. Similarly, there were no significant differences in the positions of the external cervical os, rectal ampulla, or the levator ani hiatus area during Valsalva across any of the groups (*P* > .05).

### 3.4. Multivariate linear regression analysis of factors associated with pelvic floor muscle electrophysiological parameters

Based on the results of preliminary univariate analyses, multiple linear regression models were constructed for each pelvic floor electromyographic parameter significantly associated with DRA classification. Independent variables included DRA classification, demographic and clinical characteristics such as age, height, mode of delivery, parity and other relevant covariates. For the external anal sphincter, although univariate analysis indicated that ECP was significantly lower in the non-DRA group, this association was no longer significant after adjusting for covariates (β = −8.745 μV, *P* = .304, 95% CI: [−25.509, 8.020]). Mode of delivery was an independent predictor. In comparisons between the mild and moderate-to-severe DRA groups, DRA classification remained an independent predictor of ARP in the puborectalis (β = 9.344 μV, *P* = .018, 95% CI: [1.659, 17.030]), pubococcygeus (β = 8.601, *P* = .035, 95% CI: [0.621, 16.580]), and urethral sphincter (β = 7.903 μV, *P* = .015, 95% CI: [1.593, 14.213]). Additionally, mode of delivery and parity were consistently identified as significant covariates across the models. In contrast, DRA classification was not an independent predictor of vaginal sphincter ARP (β = 4.741, *P* = .154, 95% CI: [‐1.809, 11.290]) (Table 3).

**Table 3:** Four separate multivariable linear regression analyses of DRA severity on ARP values of pelvic floor muscles.

Muscle group (dependent variable)	β for DRA severity (μV)	95% CI	*P*
Puborectalis	9.344 (μV)	1.659–17.030	.018
Pubococcygeus	8.601 (μV)	0.621–16.580	.035
Vaginal sphincter	4.741 (μV)	‐1.809 to 11.290	.154
Urethral sphincter	7.903 (μV)	1.593–14.213	.015

Each model adjusted for age, height, delivery mode, parity, and other relevant covariates.

ARP = anterior resting potential, DRA = diastasis recti abdominis.

### 3.5. Correlation analysis of maximum inter-rectus distance with multisite myoelectrophysiology of pelvic floor muscle

**Figure 2.**
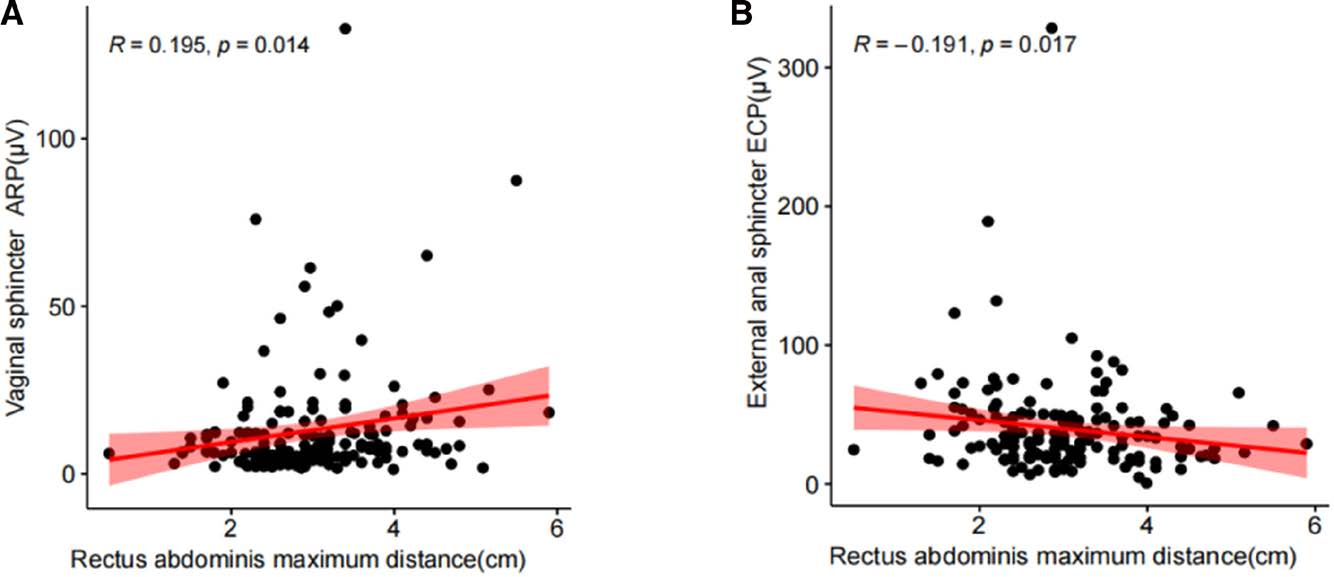
Correlation between maximum inter-rectus distance and pelvic floor muscle EMG. (A) Shows the Spearman rank correlation analysis of maximum inter-rectus distance with vaginal sphincter ARP; (B) Shows the Spearman rank correlation analysis of maximum inter-rectus distance with external anal sphincter ECP.

The relationship between the maximum rectus abdominis distance and the electromyographic parameters of 6 pelvic floor muscles (puborectalis, pubococcygeus, iliococcygeus, vaginal sphincter, urethral sphincter, and external anal sphincter) was analyzed using Spearman rank correlation. The results demonstrated a weak positive correlation between rectus abdominis distance and the ARP of the vaginal sphincter (*R* = 0.195, *P* < .05), and a weak negative correlation with the ECP of the external anal sphincter (*R* = −0.191, *P* < .05) (Fig. 2).

## 4. Discussion

Pregnancy and childbirth are recognized as major risk factors for both DRA and PFD. With the advancement of rehabilitation medicine, particularly the increasing systematic understanding of abdominopelvic dynamics, the relationship between core abdominal muscles and the pelvic floor has become a major research focus.^[25,26]^ Spitznagle et al^[8]^ reported that decreased abdominal muscle strength may impair co-contraction of the pelvic floor muscles, thereby increasing the risk of PFD. However, other studies found no significant association between these 2 conditions.^[27,28]^ This study employed multisite EMG and three-dimensional pelvic floor ultrasound to evaluate the impact of DRA on pelvic floor muscle activity and anatomy in postpartum women, aiming to support precision rehabilitation strategies.

Baseline comparisons revealed no significant differences between the DRA and non-DRA groups in terms of age, body mass index, delivery mode, parity, gestational weight gain, or macrosomia rate (*P* > .05). However, previous studies have reported associations between DRA and factors such as maternal age, gestational weight gain, multiple pregnancies, cesarean delivery, and parity.^[29,30]^ This discrepancy may result from the high proportion of mild DRA cases (nearly 50%) in the current sample, whose characteristics closely resemble those of the non-DRA group, possibly obscuring differences across the cohort. The DRA incidence in this study was 88.5%, slightly higher than reported in some domestic studies (79.7%).^[31]^ This may be attributed to the inclusion of multiparous women.

To assess the impact of DRA on pelvic floor anatomy, three-dimensional ultrasound was used to evaluate positional changes in the bladder neck, cervical os, and rectal ampulla before and after Valsalva, along with measurements of the levator hiatus area. No significant differences were found between the DRA and non-DRA groups, nor between the mild and moderate-to-severe DRA subgroups (*P* > .05). These results are consistent with the findings reported by Zhu and Qu.^[32,33]^ These observations suggest that DRA exerts limited influence on pelvic support structures in the early postpartum period and is unlikely to result in overt organ prolapse or anatomical abnormalities.

In terms of pelvic floor electromyography, there were no substantial overall differences in EMG parameters between the DRA and non-DRA groups. Although preliminary univariate analysis showed that the ECP of the external anal sphincter was significantly lower in the DRA group than in the non-DRA group, multiple linear regression analysis revealed that DRA was not an independent predictor of the ECP after adjusting for demographic and clinical characteristics in a multivariate regression model. Further multivariate regression analysis within the DRA subgroups indicated that DRA severity independently predicted ARP in the puborectalis, pubococcygeus, and urethral sphincter muscles. These findings suggest that moderate-to-severe DRA may affect the resting-state electrophysiological activity of these muscles by altering pelvic floor muscle tone. Moreover, mode of delivery and parity emerged as significant covariates, highlighting the need to account for obstetric factors in clinical decision-making. Similarly, Zhu et al reported higher resting EMG values in women with DRA compared to controls.^[32]^ Wu et al further observed that EMG changes may precede the onset of clinical symptoms, highlighting its value in early functional assessment.^[34]^ Given the critical role of the rectus abdominis in maintaining intra-abdominal pressure, trunk stability, and pelvic support, its separation may disrupt the coordination between abdominal and pelvic muscle groups, leading to compensatory tension in the pelvic floor muscles.^[25]^ Although not accompanied by anatomical abnormalities, these EMG alterations may serve as early indicators of PFD and should be considered in clinical evaluation. Notably, although vaginal sphincter ARP increased with DRA severity in univariate analysis, this association was not significant in multivariate analysis, suggesting that muscle tone is more likely influenced by external factors, such as delivery mode, rather than DRA alone.

In addition, this study identified weak correlations between the maximum inter-rectus abdominis distance and both the ARP of the vaginal sphincter and the ECP of the external anal sphincter. Our previous research similarly revealed a weak negative association between DRA and reduced ECP in specific pelvic floor muscles.^[35]^ Although these correlations reached statistical significance, the effect sizes were minimal and not sufficient to independently predict or explain clinical alterations in pelvic floor muscle function.

In clinical practice, pelvic floor rehabilitation often emphasizes strength training, such as Kegel exercises. However, this study suggests that some pelvic floor muscles in patients with moderate-to-severe DRA may exhibit increased resting tone. If intensive training is applied indiscriminately, it may further exacerbate muscle tension. Therefore, rehabilitation for these patients should prioritize relaxation and coordination training, supplemented by rectus abdominis function restoration, to help reestablish abdominopelvic synergy and reduce pelvic floor load at its source.^[36,37]^ Moreover, existing studies have shown that combining abdominal and pelvic floor muscle training is more effective in improving DRA, highlighting the importance of integrated rehabilitation strategies.^[36]^

Although this study offers valuable insights into the early effects of DRA on pelvic floor electromyography, several limitations must be noted. First, it was a single-center retrospective study with a relatively small sample size. The recruitment period also overlapped with the peak incidence of DRA in the early postpartum phase, contributing to group imbalance. Furthermore, all participants were assessed between 6 and 12 weeks postpartum, and the absence of long-term follow-up data limits the assessment of DRA’s prolonged impact on pelvic floor function. Future studies should adopt large-sample, multicenter, and prospective designs across different postpartum stages to clarify the relationship between DRA and PFD and to guide more effective intervention strategies.

## 5. Conclusion

DRA has limited impact on pelvic anatomical structures in the early postpartum period, but its severity significantly influences muscle tone-related EMG parameters in specific pelvic floor muscles, particularly the puborectalis, pubococcygeus, and urethral sphincter. Rehabilitation strategies should be tailored according to DRA severity and individualized factors, such as delivery mode and parity, to enhance the effectiveness of postpartum recovery.

## Author contributions

**Conceptualization:** Feifei Fu.

**Data curation:** Feifei Fu, Hongyun Zhang.

**Formal analysis:** Na Zhang.

**Investigation:** Shuang Zhang, Hongyun Zhang.

**Methodology:** Shuang Zhang, Zhangbiao Xuan.

**Resources:** Zhenwei Xie.

**Software:** Feifei Fu.

**Supervision:** Zhangbiao Xuan, Na Zhang, Zhenwei Xie.

**Validation:** Shuang Zhang.

**Writing – original draft:** Feifei Fu.

**Writing – review & editing:** Feifei Fu, Zhenwei Xie.
